# Case Report: Uroenteric Fistula in a Pediatric-en-bloc Kidney Transplant Manifests as Deceptive Watery Diarrhea and Normal Anion Gap Acidosis

**DOI:** 10.3389/fped.2021.687396

**Published:** 2021-07-12

**Authors:** Malek Al Barbandi, Marissa J. Defreitas, Juan C. Infante, Mahmoud Morsi, Patricia A. Arroyo Parejo Drayer, Chryso P. Katsoufis, Wacharee Seeherunvong, Jayanthi Chandar, George W. Burke, Carolyn L. Abitbol

**Affiliations:** ^1^Division of Pediatric Nephrology, Department of Pediatrics, University of Miami/Holtz Children's Hospital, Miami, FL, United States; ^2^Division of Kidney/Pancreas Transplant, Department of Surgery, Miami Transplant Institute, University of Miami/Jackson Memorial Hospital, Miami, FL, United States; ^3^Department of Radiology (Voluntary), University of Miami/Jackson Memorial Hospital, Miami, FL, United States; ^4^Department of Radiology, Nemours Children's Hospital/University of Central Florida, Orlando, FL, United States

**Keywords:** uroenteric fistula, pediatric-en-bloc transplant, non-anion gap acidosis, urinary diarrhea, CFTR-SLC26

## Abstract

**Introduction:** The diagnosis of a post–surgical uroenteric fistula can be challenging and may be delayed for months after symptoms begin. A normal anion gap metabolic acidosis has been reported in up to 100% of patients after ureterosigmoidostomy, and bladder substitution using small bowel and/or colonic segments. Here, we describe a rare case of a pediatric patient who developed a uroenteric fistula from the transplant ureters into the small bowel, after an en-bloc kidney transplantation resulting in profound acidosis and deceptive watery diarrhea.

**Case Presentation:** The patient is an 8-year-old girl with end stage kidney disease (ESKD) secondary to focal segmental glomerulosclerosis. Through a right retroperitoneal approach, she underwent a right native nephrectomy and a pediatric deceased donor en-bloc kidney transplant including two separate ureters. One month later, she had a renal allograft biopsy for suspected rejection. During the week after the biopsy, she experienced abdominal pain followed by watery diarrhea and metabolic acidosis requiring continuous bicarbonate/acetate infusions. An extensive gastro-intestinal evaluation for the cause of the diarrhea including endoscopy was inconclusive. The urine output decreased to <500 ml daily; although, the kidney function remained normal. After 2 weeks of unexplained watery diarrhea a magnetic resonance urogram with contrast was performed which demonstrated extravasation of urine from both ureters with fistulization into the small bowel. She underwent corrective surgery which identified the fistulous tract, which was resected and both ureters were re-implanted. The diarrhea and acidosis resolved, and she has maintained normal renal allograft function for over 1 year.

**Conclusion:** An important aspect in the early diagnosis of a uroenteric fistula is the sudden onset of severe hyperchloremic metabolic acidosis that results when urine is diverted into the intestinal tract. The mechanism is similar to that described in cases of urinary diversions and/or bladder augmentation using the intestine. Important diagnostic tools are the measurements of solute excretion and pH in the urine as compared to the “watery diarrhea” or bowel output.

**Summary:** We describe a case of a uroenteric fistula in a pediatric-en-bloc kidney transplant patient that went undiagnosed for almost 3 weeks due to the deceptive nature of the watery diarrhea which was actually urine. A uroenteric fistula should be considered in the differential diagnosis of diarrhea and hyperchloremic metabolic acidosis as a complication of kidney transplant. The simultaneous comparison of stool and urine pH and solute excretions may lead to the diagnosis, appropriate imaging and surgical intervention.

## Introduction

Uroenteric fistula represents an abnormal communication between the urinary and enteral tracts. Although such fistulas are uncommon, they cause significant morbidity and may markedly affect the patient's quality of life. Uroenteric fistulas most frequently occur as a consequence of abdominal malignancies, traumatic or iatrogenic injuries (1, 2). Untreated fistulas can lead to serious complications, including peritonitis, sepsis, malabsorption, electrolyte abnormalities, acute kidney injury, metabolic acidosis, and hyperammonemia (1, 2). Here, we describe the rare case of a patient who developed a urinoma following pediatric-en-bloc kidney transplant with extravasation of urine from both ureters and fistulization into the small bowel. This resulted in severe hyperchloremic metabolic acidosis and persistent deceptive watery diarrhea.

## Case Presentation

**Figure 1:**
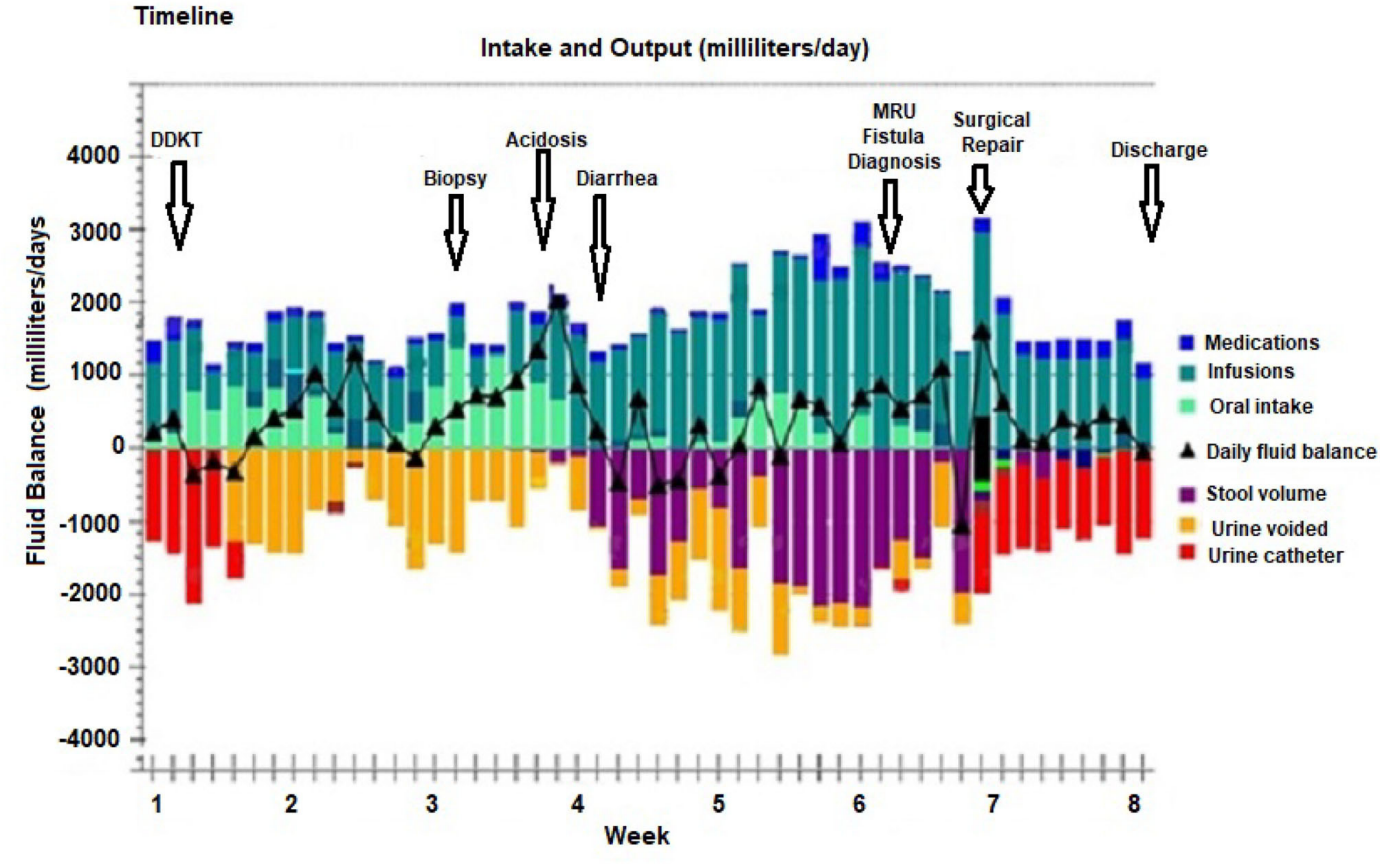
Timeline from deceased pediatric donor en-bloc kidney transplant (DDKT), renal allograft biopsy with subsequent development of uroenteric fistula which manifested as normal anion gap acidosis and watery diarrhea which was diagnosed by magnetic resonance imaging urogram (MRU), followed by surgical repair and discharge after 8 weeks of hospitalization. The daily fluid balance with urine and stool volumes are color coded.

An 8-year-old girl with a history of end stage kidney disease (ESKD) secondary to steroid resistant nephrotic syndrome and focal segmental glomerulosclerosis (FSGS), underwent right native nephrectomy, with a pediatric-en-bloc deceased donor kidney transplant. The transplant operation was performed in a retro-peritoneal fashion. The right native kidney was excised to create space for the two en-bloc pediatric kidneys. The arterial anastomosis was performed in an end-to-side fashion between the distal donor aorta and the distal recipient aorta. The venous anastomosis was performed in an end-to-side fashion between the inferior aspect of the donor inferior vena cava (IVC) and the distal recipient IVC. The bladder was small, but of sufficient size to permit the placement of two separate extravesicular (Lich-Gregoire technique) uretero-vesical anastomoses, performed without tension, and without stents. Her post-operative course was complicated by recurrence of FSGS manifesting as heavy proteinuria within 24 h following the transplant. She was treated with therapeutic plasma exchange and rituximab with resolution of the proteinuria. Four weeks after the transplant, she suffered refractory abdominal pain with fever and elevated serum creatinine. Serial abdominal ultrasounds showed resolving postoperative perinephric collections around each kidney with stable moderate hydronephrosis of the lateral kidney. She underwent a kidney biopsy for suspected rejection. The biopsy revealed borderline T-cell mediated rejection which was treated with solumedrol given as a rapid taper over 10 days from 300 to 5 mg for a total dose of 750 mg. Although the fever resolved and the abdominal pain improved, she developed watery diarrhea (1–2 liters daily without mucus or blood) with a hyperchloremic normal anion gap metabolic acidosis. Clinical examination revealed abdominal distension with generalized tenderness. Abdominal ultrasound showed a new complex collection involving the pouch of Douglas measuring 6.8 ×6.4 ×3.3 cm. As a result, she was started on antibiotics for possible peritonitis. The clinical course timeline is depicted in Figure 1.

## Diagnostic Assessment

A comprehensive screening for diarrhea included: stool culture for enteropathogenic bacteria, viral tests, ova and parasites, *Clostridium difficile*, and Celiac disease serologies which were negative. Upper and lower endoscopies were normal. From the onset of the watery diarrhea, she manifested severe hyperchloremic metabolic acidosis (serum chloride 112 and bicarbonate 6–9 mmol/L) with normal anion gap (Table 1). Over the course of 3 weeks, the diarrhea worsened and urine output decreased to <500 ml daily although the serum creatinine remained normal at 0.3 mg/dL. Her stool became increasingly clear and watery as she was maintained on bowel rest and total parenteral nutrition. She required up to 10–12 mmol/kg/day of bicarbonate/acetate to correct her metabolic acidosis. To evaluate for a uroenteric fistula, the stool and urine pH and solutes were evaluated concurrently (Table 1). Notably, the analysis of the solutes from the bowel and bladder indicated that the bowel output was extremely alkaline with a pH of 9.0. Moreover, there was indication that there was reabsorption of chloride and ammonium from the bowel and secretion of bicarbonate into the bowel (Table 1). This was in the setting of no enteral feedings or medications.

**Table 1:** Comparison of blood, bowel and bladder solutes during hyperchloremic acidosis induced by uroenteric fistula.

	**Blood acid/Base balance**		**Bowel**	**Bladder**
	**Acidosis**	**Normo-acidemia**	**Watery stool**	**Urine**
pH	**7.20**	7.36	**9.0**	6.0
Sodium mmol/L	137	136	98	67
Potassium mmol/L	4.4	4.2	5.7	3.0
Chloride mmol/L	**116**	110	31	48
HCO3^−^ mmol/L	**7**	20		
Serum anion gap (mmol/L) (Normal 6–22)	18	10		
Creatinine mg/dL	0.29	0.29	**17.65**	**5.44**
Osmolarity (mOsm/L)	273	273	242	131
Total protein mg/dL	5.1	5.6	**55**	**14**
Albumin mg/dL	**2.9**	**3.2**	**3.3**	** <1.2**
Urine Pr/Cr (mg/mg) (Normal <0.2)			**3.11**	**2.57**
Urine alb/cr (mg/g) (Normal <30)			**187**	**220**
Urine anion gap (HCO3)			**+72.7**	+22
Urine osmolar gap (NH4)			**+134**	+61
FeNa%			**1.6%**	**3.6%**
FeK%			**2.0%**	**3.5%**
FeCl%			**0.58%**	**2.9%**
Tubular reabsorption of Chloride %			**99.4%**	**97.1%**

*Abnormal values are in bold. mmol/L, millimoles/liter; mOsm/L, milliosmoles/ liter; mg/dl, milligrams/deciliter; U Pr/Cr, Urine Protein/Creatinine ratio (Nl <0.2 mg/mg; Nephrotic >2.0 mg/mg); Ualb/cr, Urine albumin/creatinine ratio (Nl <30 mg/g; nephrotic > 1,000 mg/g). HCO3, bicarbonate; NH4, ammonium; FeNa%, Percent fractional excretion of sodium; FeK%, Percent fractional excretion of potassium; FeCl%, Percent fractional excretion of chloride. Please see the schematic in **Figure 3** (3, 4)*.

**Figure 2:**
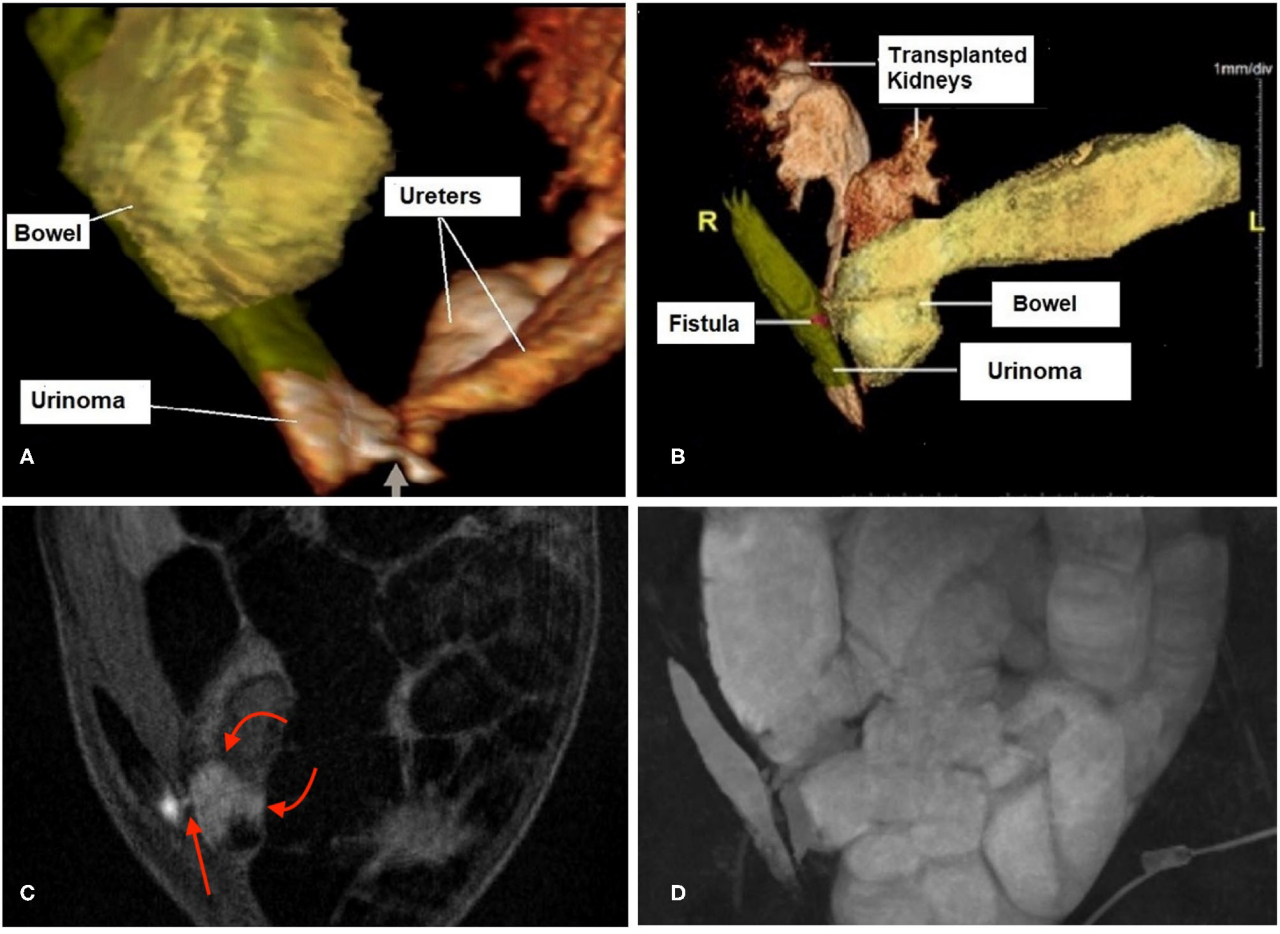
3D magnetic resonance urogram (MRU) images show a urinoma to small bowel fistula. **(A)** Shows the 2 ureters of the pediatric-en-bloc kidney transplant draining into a 8.6 × 3.9 × 1.1 cm fluid collection (labeled “Urinoma”) located in the right lateral abdominal wall. The connection between the distal ureters at the base of the collection is shown (short arrow). **(B)** Shows the urinoma has developed a fistulous tract (magenta highlight labeled “Fistula”) which drains into the proximal small bowel limb where the excreted contrast is seen progressively filling the bowel. **(C)** This sequence shows a cross-section of the tiny fistula (red arrow). Note that the bright urine in the urinoma is seen starting to mix with the fluid in the adjacent bowel (curved arrows), in contrast to the otherwise dark bowel contents throughout the rest of the image. **(D)** This 3D maximum intensity projection shows the location of the urinoma along the right abdominal wall which is filling the markedly dilated bowel with fluid which is urine.

A magnetic resonance urogram (MRU) was performed, which demonstrated extravasation of urine from both transplanted ureters into a right lateral retroperitoneal collection. This extended upwards in between the lateral abdominal wall musculature (Figure 2). This collection communicated with the small bowel through a tiny fistulous tract.

## Therapeutic Intervention

She underwent corrective surgery in which the mid-ureters of both renal allografts were noted to be necrotic. The urinoma was drained and the fistula that involved the small bowel at the mid-jejunum was resected and repaired. The right transplant ureter was anastomosed to the right native ureter with an end-to-end anastomosis. The left transplant ureter was re-implanted directly into the bladder. The stool output normalized immediately following the surgery and the acidosis resolved; she was discharged 9 days later with stable kidney function. Ureteral stents were subsequently removed 4 weeks after the corrective surgery.

## Follow-Up and Outcomes

At the time of this writing, 20 months post-transplant and 18 months post repair of the uroenteric fistula, the patient is doing well with normal urine output and stable renal function. The parents expressed gratitude at her successful diagnosis and surgical repair and approved the reporting of the details of her case.

## Discussion

This case of a uroenteric fistula presenting as deceptive watery diarrhea in a pediatric-en-bloc kidney transplant patient provides unique lessons in the pathophysiology of the acidosis and the required diagnostic imaging. A fistula is an abnormal communication between 2 epithelium-lined cavities. Uroenteric fistulas can occur between any part of the urinary tract and the small or large bowel. Nomenclature and classification are generally based on the organ of origin in the urinary tract and the termination of the fistula in the segment of the gastrointestinal tract (1, 2). Uroenteric fistulas most frequently occur as a consequence of abdominal malignancies, inflammation (as in Crohn's Disease), trauma, or surgical diversions for bladder substitution or augmentation (1, 2). Importantly, the most common clinical manifestations of uroenteric fistulas are pneumaturia, fecaluria and urinary tract infection due to pressure gradients favoring the movement of feces into the urinary tract (1, 2).

The clinical presentation of severe acute hyperchloremic metabolic acidosis in association with deceptive watery diarrhea is a rare manifestation of a uroenteric fistula and an important lesson in its early recognition (1, 2). The only previous report in the literature was of a 46-year-old woman with an enterovesical fistula between the urinary bladder and sigmoid colon presenting with watery diarrhea for 6 months and life-threatening normal anion gap acidosis (5). In the case of the 46-year-old woman, a high pressure neurogenic bladder with fistula formation into the sigmoid colon resulted in the flow of urine into the colon with deceptive watery diarrhea and profound acidosis (5). In the present case, the unusual fistulization causing urine flow into the jejunum with continuous exposure of urine to the entire bowel manifested as “watery diarrhea” and severe hyperchloremic acidosis. As such, this case provides an opportunity to examine the pathophysiology of the profound acidosis that occurs when urine is exposed to the intestinal mucosa (6–11). Comparison of the pH and solute excretions from the bowel and the bladder, summarized in the Table 1, supports the reabsorption of chloride and secretion of bicarbonate by the bowel mucosa. There is also a positive “osmolar gap” in the bowel output (i.e., urine exposed to bowel mucosa) which supports the presence of ammonium (NH4+) (3, 4, 12).

**Figure 3:**
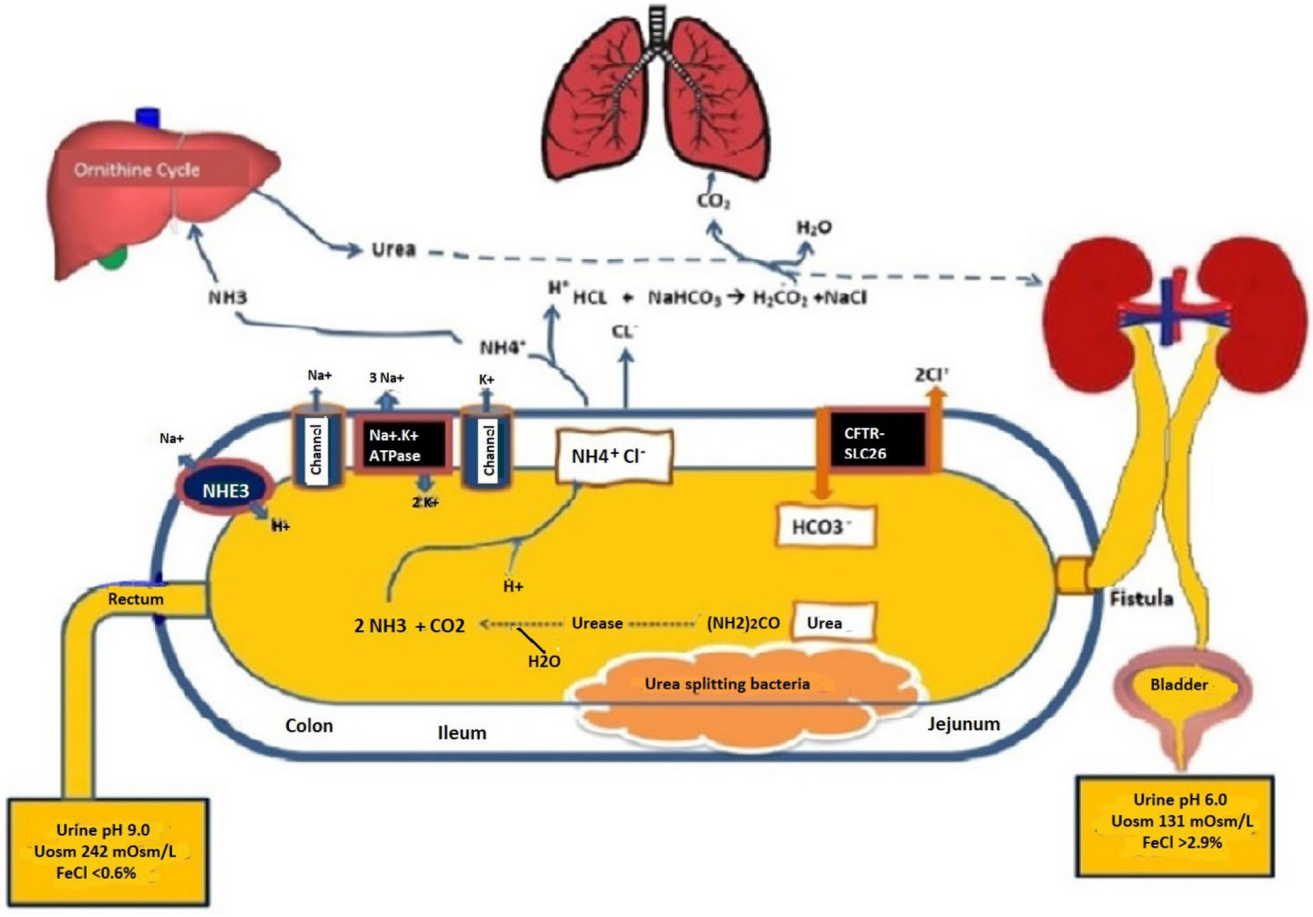
Schematic of the uroenteric fistula with differential urine pH and solutes from the bladder and bowel urines taken concurrently when in metabolic balance maintained by parenteral buffer therapy at 10–12 mmol/kg/day as sodium/ potassium acetate. H_2_O, Water; HCO_3_, Bicarbonate; NaCl, Sodium chloride; (NH_2_)_2_CO, Urea; NH_3_, ammonia; CO_2_, carbon dioxide; NH_4_Cl, ammonium chloride; CFTR-SLC26, Cystic fibrosis-chloride transporter-soluble carrier family 26; Na-K ATPase, Sodium-potassium adenosine triphosphatase; NHE3, sodium-hydrogen exchanger 3; Uosm, urine osmolarity; FeCl, Fractional excretion of chloride.

Figure 3 provides a schematic of the potential physiologic mechanisms that are involved in generating a life-threatening acidosis in the setting of uroenteric fistula. A priori, when urine is exposed to the urea-splitting organisms in the bowel lumen, the high concentrations of urea are rapidly metabolized to ammonia (NH3) and carbon dioxide (3, 4, 12). This can lead to the rapid reabsorption of free ammonia into the circulation or combined with chloride as ammonium chloride. Although the mechanism in the setting of a uroenteric fistula has not been well studied, the *cystic fibrosis transmembrane conductance regulator* and *SLC26* transporters (CFTR-SLC26) must play a prominent role in the secretion of bicarbonate into the bowel lumen with reabsorption of chloride (13, 14). Concurrent anion channels including the sodium-hydrogen exchanger 3 (NHE3) are found on the apical enterocytes of the intestine as well as on the basolateral cells. These may also contribute to the release of bicarbonate into the intestinal lumen (15).

Many of these metabolic complications are influenced by the degree to which solute absorption occurs across the bowel segment. The factors that influence solute absorption include: the segment of bowel involved, the surface area of the bowel exposed, time of retention of urine, concentration of solutes in the urine, renal function, and pH and osmolarity of the fluid (10–15). These factors influence the amount of solutes absorbed and, as such, influence the type of metabolic complications and their severity. The duration that the intestinal segment has been exposed to the urinary tract has also been suggested as a determinant of solute absorption. It has been suggested that the activity of the transport processes diminishes with time (10–12). In analyzing the pH and solutes in the urine from the bladder and the bowel in our patient, it was apparent that bicarbonate secretion was predominant with the suggestion that ammonium (NH4+) was also in high production. The reabsorption of chloride with both ammonium and sodium was consistent with our patient's solute excretions and plasma electrolytes (3, 4).

In our case we confirmed the diagnosis via MRU with contrast, which showed the extravasation of urine from the ureters and the fistulous communication between the ureters and small bowel. The development of mid-ureteral necrosis of both ureters is consistent with an ischemic injury to both pediatric-en-bloc kidneys resulting in insufficient flow from both the renal arteries to the ureters, while the distal ureters were revascularized and protected by inflow from the bladder, resulting in bilateral mid-ureteral necrosis and subsequent urine leak. The tiny fistulous tract to the small bowel may have occurred inadvertently during the kidney transplant biopsy procedure. The high dose steroids given for the suspected rejection may have masked some of the symptoms associated with the development of the urinoma.

Although some fistulas heal with conservative management, surgery is often necessary (1, 2). The location and underlying disorder often determine the symptoms. Imaging with contrast in the bowel and intravenous contrast to outline the urinary tract in cross-sectional imaging with a computed tomography (CT) scan or MRU often helps in the diagnosis. The location and underlying disorder often determine the symptoms. The management involves resection of the bowel segment, stenting the ureter, and (rarely) placing a nephrostomy tube (1, 2, 5). A nephroureterectomy would only be indicated if the kidney were non-functioning. Our patient had corrective surgery with successful reimplantation of both transplanted ureters and resection of the fistula and involved small bowel with total recovery of urinary flow and resolution of acidosis.

## Conclusion

This case has provided insight into the pathophysiologic mechanisms of hyperchloremic metabolic acidosis in patients with uroenteric conduits which may facilitate long term management in urologic patients. Ultimately, a surgical repair in our patient was required. The simultaneous comparison between the pH and solutes from the bowel and bladder supported the suspicion of uroenteric fistula leading to the imaging studies necessary to determine the surgical approach.

## Data Availability Statement

The original contributions generated for the study are included in the article/supplementary material, further inquiries can be directed to the corresponding author/s.

## Ethics Statement

Ethical review and approval was not required for the study on human participants in accordance with the local legislation and institutional requirements. Written informed consent to participate in this study was provided by the participants' legal guardian/next of kin. Written informed consent was obtained from the minor(s)' legal guardian/next of kin for the publication of any potentially identifiable images or data included in this article.

## Author Contributions

All authors participated in the clinical care of the patient during the course of the hospitalization. All authors contributed to the article and approved the final manuscript.

## Conflict of Interest

The authors declare that the research was conducted in the absence of any commercial or financial relationships that could be construed as a potential conflict of interest.
